# Inverse association between obesity and suicidal death risk

**DOI:** 10.1186/s12888-024-06381-z

**Published:** 2025-01-08

**Authors:** Joonyub Lee, Seung-Hwan Lee, Mee-Kyoung Kim, Hyuk-Sang Kwon, Jae-Seung Yun, Yeoree Yang, Kun-Ho Yoon, Jae-Hyoung Cho, Chi-Un Pae, Kyungdo Han, Jang Won Son

**Affiliations:** 1 Division of Endocrinology and Metabolism, Department of Internal Medicine, Seoul St. Mary’s Hospital, College of Medicine, The Catholic University of Korea, Seoul, Republic of Korea; 2 Division of Endocrinology and Metabolism, Department of Internal Medicine, Yeouido St. Mary’s Hospital, College of Medicine, The Catholic University of Korea, Seoul, Republic of Korea; 3 Division of Endocrinology and Metabolism, Department of Internal Medicine, College of Medicine, St. Vincent’s Hospital, The Catholic University of Korea, Seoul, Republic of Korea; 4 Department of Psychiatry, College of Medicine, The Catholic University of Korea, Seoul, Korea; 5 Cell Death Disease Research Center, College of Medicine, The Catholic University of Korea, Seoul, Korea; 6 Department of Statistics and Actuarial Science, Soongsil University, 369 Sangdo-ro, Dongjak-gu, Seoul, 06978 Republic of Korea; 7 Division of Endocrinology and Metabolism, Department of Internal Medicine, Bucheon St. Mary’s Hospital, College of Medicine, The Catholic University of Korea, 327 Sosa-ro, Bucheon, 14647 Republic of Korea

**Keywords:** Suicide, Completed, Obesity, Body mass index, Depressive disorder, Major

## Abstract

**Background:**

Suicide is a significant yet preventable public health issue. Body mass index (BMI) is a readily measurable indicator associated with various health outcomes. However, the relationship between BMI and suicidal death risk is complex and warrants further investigation, particularly within contemporary, non-Western contexts with consideration of potential confounders. The purpose of this study was to investigate the relationship between BMI and the risk of suicidal death.

**Methods:**

This study was nationwide, retrospective, observational study based on Korean National Health Insurance Service database. We analyzed 4,045,081 participants who were aged > 19 years and underwent national health surveillance in 2009. The participants were categorized according to their BMI (underweight: < 18.5 kg/m², normal weight: 18.5–23 kg/m², overweight: 23–25 kg/m², class I obesity: 25–30 kg/m², and class II obesity: > 30 kg/m²). The primary outcome was the death events caused by suicide which was defined by International Classification of Disorders (ICD-10) codes (X60–X84) and death records documented by the Korea National Statistical Office. Multivariate Cox proportional hazard regression analysis was performed to estimate the risk of suicidal death with respect to BMI categories after adjusting for potential confounders (age, sex, income, diabetes, hypertension, dyslipidemia, smoking, drinking, exercise, self-abuse, waist circumference, schizophrenia, bipolar disorder, eating disorder, cancer, anxiety, and substance use disorder).

**Results:**

Underweight individuals had an increased risk (hazard ratio [HR] 1.44, 95% confidence interval [CI] 1.31–1.57) while overweight (HR 0.79, 95% CI 0.76–0.83), class I (HR 0.76, 95% CI 0.71–0.80) and class II obesity (HR 0.71, 95% CI 0.63–0.81) were associated with decreased risks of suicidal deaths compared to those of the normal weight individuals (BMI 18.5–23). This trend was consistent regardless of the presence of major depressive disorder (MDD) or the type of living arrangements of the participants.

**Conclusions:**

Suicidal death risk was inversely correlated with BMI categories, independent of MDD or living arrangements. Our data suggests the importance of physiological factors associated with body mass in understanding suicidal death risk. Furthermore, these data provide valuable insights to where the public health resources should be invested to reduce suicidal death rates.

**Supplementary Information:**

The online version contains supplementary material available at 10.1186/s12888-024-06381-z.

## Background

Suicide is a significant yet preventable public health challenge, which imposes a considerable health burden in many countries. Suicide is the leading cause of death among young adults in many countries and accounts for more than 0.7 million deaths annually worldwide [1, 2]. Despite community-based and primary care-based approaches have demonstrated effectiveness in preventing suicide, the incidence of suicide remains substantial [3–5]. To maximize the effectiveness of suicide prevention program, it is important to identify the subpopulations who has a high potential for suicidal deaths.

Previous studies have suggested that genetic, social, and physical factors may associate with a risk for suicide [6, 7]. Single-nucleotide polymorphisms in genes related to serotonergic signaling (Htr1a, Htr2a, Htr5a, Tph1, and Slc6a4) have been linked to suicide risk [8]. Other factors, such as single-person house hold, introverted disposition, exposure to traumatic events, and financial crises have also been associated with an increased risk of suicide [6, 9]. Concomitant chronic medical and mental illnesses such as major depressive disorder (MDD), migraine, sleep apnea, and insomnia was suggested to associate with a higher risk of suicide [10, 11]. However, most of these variables are often unobtainable in general population and therefore may not be appropriate to be used for health administrative purposes.

Body mass index (BMI), an easily measurable parameter, is associated with various health outcomes, including mental health issues and suicidal behaviors [12]. The relationship between BMI and suicide risk, however, is complex. High BMI is often accompanied by social stigma, discrimination, and economic difficulties, which, along with associated mental health conditions like MDD [13], can increase the risk of suicidal attempts or deaths [12, 14]. Although there is evidence to suggest a higher incidence of suicide among individuals with obesity [15–18], some studies propose that obese individuals may have a lower risk of suicide [18–22]. These complexing previous studies, primarily based on outdated data from Western populations, demand the need for an in-depth examination of the BMI-suicidal death risk relationship, with consideration of potential confounding factors.

To address this issue, our study investigates the relationship between BMI and the risk of suicidal death, paying particular attention to potential confounding factors, within a contemporary, non-Western setting. We conducted an analysis using a national cohort from South Korea, a country currently facing a rapid and unprecedented rise in both suicide rates and obesity prevalence.

## Methods

### Data

We collected data from the National Health Information Database (NHID), a Database in South Korea containing health examination results and insurance claims data. The NHID, which ensures coverage for virtually all Koreans aged 40 and above and employees over the age of 20, is founded upon the National Health Insurance Service (NHIS), a government-owned, single-payer organization [23, 24]. The information contained in this dataset includes lifestyle behavior, blood test results, anthropometric measures, treatment details, records of disorders diagnosed using International Classification of Disorders (ICD-10) codes, and questionnaire responses about lifestyle behavior. On request, the review board reviewed the study proposal and provided de-identified data to the researchers.

### Study population

From the NHIS database, we retrospectively enrolled 4,234,415 participants aged > 19 years who underwent national health surveillance in 2009. Among these participants, 179,183 with missing values and 10,151 lost to follow-up for more than a year were excluded and final number of 4,045,081 participants were analyzed (Additional file: Figure S1). This study population was followed up from baseline to the date of death or until December 31, 2021, whichever came first. The median follow-up period was 11.31 years (interquartile range: 11.11–11.57).

### Definition and measurements

The primary outcome of this study was the death events caused by suicide. Completed suicide was defined as when the participants met the criteria of both International Classification of Diseases 10th Revision (ICD-10) codes (X60–X84) and death records documented by the Korea National Statistical Office.

To define the weight class, we followed the World Health Organization Asia-Pacific region criteria [25]. The cutoff values were as follows:

- <18.5 kg/m2: underweight.
- 18.5–23 kg/m2: healthy weight.
- 23–25 kg/m2: overweight.
- 25–30 kg/m2: class I obesity.
- >30 kg/m2: class II obesity.

Abdominal obesity was defined according to the participants’ waist circumference following the guideline of Korean Society for the Study of Obesity (Men ≥ 90 cm, women ≥ 85 cm) [26]. The presence of MDD was defined when the participants had the history of claiming ICD-10 codes F32 or F33. Schizophrenia was defined as having a record of claiming ICD-10 codes F20–F29 within 1 year. Bipolar disorder was defined as having a record of claiming ICD-10 codes F30, F31, or F34.0 within 1 year. Eating disorder was defined as having a record of claiming ICD-10 code F50 within 1 year. Anxiety disorder was defined as having a record of claiming ICD-10 codes F40 or F41 within 1 year. Substance use disorder was defined as having a record of claiming ICD-10 codes F10–F19 within 1 year. Any solid cancer was defined as having a record of claiming ICD-10 C codes within 1 year. Diabetes mellitus was defined when the participants the met the criteria of both ICD-10 codes (E10, E11–14) and the prescription code of anti-diabetic medications. Hypertension was defined when the participants met the criteria of ICD-10 codes (I10-13 or I15) and had the history of antihypertensive medication prescription or a systolic/diastolic blood pressure ≥ 140/90 mmHg. Dyslipidemia was defined when the participants had the claim of ICD-10 code E78, with at least one prescription claim per year for lipid-lowering drugs or a total cholesterol level ≥ 240 mg/dL. The estimated glomerular filtration rate (eGFR) was estimated by the Modification of Diet in Renal Disease formula. The presence of chronic kidney disease was defined when the individual’s eGFR was lower than 60 mL/min/1.73 m^2^. Current smoking habits, alcohol consumption, and exercise frequency data were determined by a questionnaire sourced from the health examination database. Alcohol consumption was stratified into two groups: <30 g/day and ≥ 30 g/day. Regular exercise was defined as engaging in strenuous physical activity for over 20 min at least three times per week or moderate physical activity for over 30 min at least five times per week. Household income level was dichotomized, with the lower 20% categorized as recipients of medical aid. The participants underwent overnight fasting before collecting blood samples to measure blood glucose and serum cholesterol levels. The health surveillance examination was conducted in healthcare facilities which were certified from the NHIS.

### Statistical analyses

The baseline characteristics between BMI categories were compared by Student’s t-test (continuous variables) or χ2 test (categorical variables). The incidence rates of suicidal deaths were estimated by dividing the number of new cases by the entire duration of follow-up, measured in person-years. Multivariable Cox proportional hazard regression analysis was performed to estimate the risk of suicidal deaths among the study population. The hazard ratios (HRs) and 95% confidence intervals (CIs) was calculated after adjusting for potential confounding variables. Model 1 was estimated without adjustment. Model 2 was estimated after adjusting for age and sex. Model 3 was further adjusted for income, diabetes, hypertension, dyslipidemia, smoking, drinking, exercise, and self-abuse on Model 2. Model 4 was further adjusted for waist circumference on model 3. Model 5 was further adjusted for schizophrenia, bipolar disorder, eating disorder, cancer, anxiety and substance use disorder on Model 4. We conducted subgroup analyses to further evaluate the potential impact of key clinical parameters, including age, sex, MDD, living arrangements, smoking, drinking, exercise, cancer, schizophrenia, bipolar disorder, eating disorder, anxiety disorder, and substance use disorder. Additionally, multiple regression analysis was performed to better understand the influence of each variable on the risk of suicidal death. To assess correlations among variables, Variance Inflation Factor was calculated, indicating minimal multicollinearity (S1 Table). To represent the incidence probabilities of suicidal deaths, we performed a log-rank test after plotting Kaplan–Meier curves. SAS version 9.4 (SAS Institute, Inc., Cary, NC, USA) was used for the statistical analyses, and a two-sided P value less than 0.05 was regarded as statistically significant.

## Results

### Baseline characteristics of participants

The study participants were categorized based on their BMI categories as described in the method section. Table 1 presents the baseline characteristics of the study participants, categorized by BMI. The largest group of participants was in the normal weight category (*n* = 1,573,660), followed by class I obesity (*n* = 1,180,895), overweight (*n* = 998,895), underweight (*n* = 147,834), and class II obesity (*n* = 143,797). Baseline characteristics of participants varied significantly among each groups. The underweight group tended to have a lower average age (40.53 ± 16.63 years), a lower prevalence of MDD (3.03%) a higher rate of regular exercise (9.7%), single-person household (38.47%), and a higher prevalence of comorbid conditions, such as diabetes mellitus (3.47%), hypertension (9.28%), dyslipidemia (5.51%), and chronic kidney disease (5.64%). The underweight group comprised predominantly females (66.74%) and had a higher proportion of nonsmokers (70.38%) and nondrinkers (56.88%). There was no difference in self-harm history among each BMI groups.

**Table 1:** Baseline characteristics of study participants

BMI		− 18.5	18.5–23	23–25	25–30	30 -	*P* value
*n*		147,834	1,573,660	998,895	1,180,895	143,797	
Age		40.53 ± 16.63	45.28 ± 14.38	48.67 ± 13.4	49.08 ± 13.28	46.24 ± 13.88	< 0.0001
Age							< 0.0001
	< 40	85,649 (57.94)	574,040 (36.48)	258,458 (25.87)	302,065 (25.58)	51,786 (36.01)	
	40–64	43,032 (29.11)	816,739 (51.9)	603,661 (60.43)	709,990 (60.12)	74,523 (51.83)	
	≥ 65	19,153 (12.96)	182,881 (11.62)	136,776 (13.69)	168,840 (14.3)	17,488 (12.16)	
Sex, male		49,168 (33.26)	748,691 (47.58)	600,857 (60.15)	748,329 (63.37)	81,033 (56.35)	< 0.0001
Height		162.85 ± 8.12	163.42 ± 8.83	164.22 ± 9.33	164.5 ± 9.63	164.16 ± 10.59	< 0.0001
Weight		46.73 ± 5.16	56.63 ± 7.03	64.75 ± 7.5	72.58 ± 9.15	86.44 ± 12.24	< 0.0001
BMI		17.57 ± 0.78	21.14 ± 1.21	23.93 ± 0.57	26.73 ± 1.29	31.96 ± 2.19	< 0.0001
Waist circum.		66.19 ± 5.71	74.29 ± 6.29	81.21 ± 5.61	87.16 ± 6.15	96.8 ± 7.7	< 0.0001
Income							< 0.0001
	Q1	34,685 (23.46)	353,259 (22.45)	206,832 (20.71)	239,158 (20.25)	31,471 (21.89)	
	Q2	44,850 (30.34)	383,544 (24.37)	205,736 (20.6)	237,932 (20.15)	33,800 (23.51)	
	Q3	39,179 (26.5)	413,463 (26.27)	265,692 (26.6)	322,565 (27.32)	40,509 (28.17)	
	Q4	29,120 (19.7)	423,394 (26.91)	320,635 (32.1)	381,240 (32.28)	38,017 (26.44)	
MDD		4481 (3.03)	48,842 (3.1)	33,648 (3.37)	40,576 (3.44)	4948 (3.44)	< 0.0001
Schizophrenia		177 (0.12)	1574 (0.1)	1299 (0.13)	2126 (0.18)	475 (0.33)	< 0.0001
Bipolar disorder		89 (0.06)	1102 (0.07)	899 (0.09)	1417 (0.12)	302 (0.21)	< 0.0001
Eating disorder		86 (0.06)	629 (0.04)	300 (0.03)	354 (0.03)	58 (0.04)	< 0.0001
Anxiety disorder		1552 (1.05)	17,625 (1.12)	11,787 (1.18)	13,344 (1.13)	1481 (1.03)	< 0.0001
Substance use disorder		503 (0.34)	4092 (0.26)	2198 (0.22)	2598 (0.22)	273 (0.19)	< 0.0001
Cancer		1981 (1.34)	20,930 (1.33)	12,986 (1.3)	14,171 (1.2)	1467 (1.02)	< 0.0001
Single-person household		56,873 (38.47)	445,589 (28.32)	214,927 (21.52)	234,901 (19.89)	33,668 (23.41)	< 0.0001
Smoke							< 0.0001
	Non	104,041 (70.38)	1,005,366 (63.89)	568,864 (56.95)	641,015 (54.28)	81,875 (56.94)	
	Ex	10,162 (6.87)	175,826 (11.17)	165,237 (16.54)	210,904 (17.86)	19,394 (13.49)	
	Current	33,631 (22.75)	392,468 (24.94)	264,794 (26.51)	328,976 (27.86)	42,528 (29.58)	
Drink							< 0.0001
	Non	84,083 (56.88)	840,765 (53.43)	502,183 (50.27)	579,253 (49.05)	74,552 (51.85)	
	Mild	57,095 (38.62)	633,569 (40.26)	413,780 (41.42)	483,531 (40.95)	53,973 (37.53)	
	Heavy	6656 (4.5)	99,326 (6.31)	82,932 (8.3)	118,111 (10)	15,272 (10.62)	
Regular exercise		14,343 (9.7)	259,532 (16.49)	201,024 (20.12)	235,819 (19.97)	24,919 (17.33)	< 0.0001
Self-harm		6 (0)	46 (0)	29 (0)	27 (0)	5 (0)	0.6785
DM		5128 (3.47)	87,744 (5.58)	89,589 (8.97)	145,558 (12.33)	25,054 (17.42)	< 0.0001
HTN		13,725 (9.28)	254,364 (16.16)	264,751 (26.5)	430,050 (36.42)	71,449 (49.69)	< 0.0001
DYS		8144 (5.51)	185,999 (11.82)	194,522 (19.47)	298,088 (25.24)	44,356 (30.85)	< 0.0001
CKD		8342 (5.64)	95,395 (6.06)	71,470 (7.15)	92,337 (7.82)	11,388 (7.92)	< 0.0001
SBP		113.63 ± 14.06	118.67 ± 14.4	123.36 ± 14.39	126.76 ± 14.51	131.32 ± 15.29	< 0.0001
DBP		71.25 ± 9.34	73.97 ± 9.62	76.78 ± 9.67	79 ± 9.83	82.09 ± 10.53	< 0.0001
Glucose		90.91 ± 20.68	94.01 ± 21.32	97.86 ± 23.72	100.89 ± 25.62	104.9 ± 30.17	< 0.0001
Total cholesterol		177.64 ± 32.36	188.65 ± 35.06	197.64 ± 36.63	202.31 ± 37.59	206.13 ± 38.89	< 0.0001
Triglyceride		74.89 (74.71–75.07)	93.21 (93.14–93.29)	118.88 (118.75–119.01)	140.17 (140.03–140.31)	157.46 (157.01–157.91)	< 0.0001
HDL-C		63.89 ± 30.68	59.26 ± 29.16	54.86 ± 27.14	52.56 ± 26.72	51.24 ± 26.45	< 0.0001
LDL-C		98.59 ± 36.52	109.25 ± 37.99	116.21 ± 38.45	118.16 ± 39.21	119.34 ± 40.25	< 0.0001
GFR		92.95 ± 47.87	89.25 ± 44.95	86.52 ± 44.85	85.66 ± 45.17	86.86 ± 43.95	< 0.0001

All data were expressed as mean ± standard deviation, median (interquartile range), median (95% CI), or n (%). Student’s t-test (continuous variables) and χ2 test (categorical variables) were used for statistical analysis, and p-values < 0.05 was regarded as statistical significance. The baseline characteristics between individuals with respect to BMI intervals were compared

*Abbreviations BMI* Body mass index, *MDD* Major depressive disorder, *SBP* Systolic blood pressure, *DBP* Diastolic blood pressure, *CKD* Chronic kidney disease, *eGFR* estimated Glomerular Filtration Rate, *HDL-C* High-density lipoprotein cholesterol, *LDL-C* Low-density lipoprotein cholesterol, *CI* Confidence interval, *DM* Diabetes mellitus, *HTN* Hypertension, *DYS* Dyslipidemia

### The risk of suicidal death inversely correlates with BMI

**Fig. 1:**
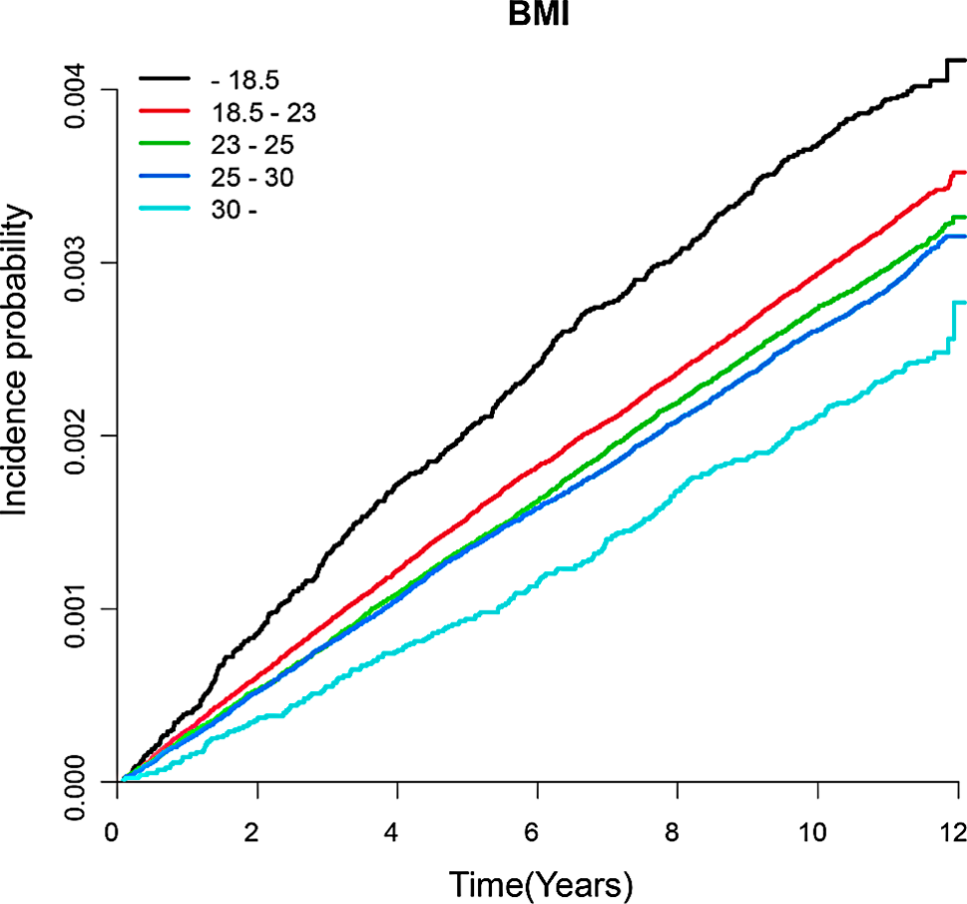
Kaplan-Meier Estimates of the Incidence of Suicidal Deaths by BMI Categories. The incidence probability of suicidal death in each BMI categories were estimated in Kaplan-Meier Curve. Black: BMI < 18.5 kg/m^2^, red: BMI 18.5–23 kg/m^2^, green: BMI 23–25 kg/m^2^, blue BMI: 25–30 kg/m^2^. light blue BMI: >30 kg/m^2^. *Abbreviation: BMI* Body mass index

During the study period (44,782,545.9 person-years), 12,509 suicidal deaths were documented. The risk of suicidal death was highest in the underweight group (HR 1.44, 95% CI 1.31–1.57), followed by the healthy weight (control) group, overweight group (HR 0.79, 95% CI 0.76–0.83), class I obesity group (HR 0.76, 95% CI 0.71–0.80), and class II obesity group (HR 0.71, 95% CI 0.62–0.81) (Table 2). This inverse correlation between BMI and suicidal death is further described in Fig. 1 using a Kaplan–Meier curve. Waist circumference is another easily obtainable parameter which associates with various health outcomes. We further investigated whether the relationship between obesity and the risk of suicidal death changes depending on the presence of abdominal obesity. Interestingly, BMI ≥ 25 kg/m² was associated with a lower risk of suicidal death, regardless of the presence of abdominal obesity (BMI ≥ 25 kg/m² without abdominal obesity HR 0.79, 95% CI 0.75–0.83; BMI ≥ 25 kg/m² with abdominal obesity HR 0.76, 95% CI 0.72–0.80, S2 Table). These data suggest that BMI is an important indicator that represents the risk of suicidal death.

**Table 2:** Changes in suicidal death risk based on BMI categories

BMI	*N*	Suicide	Duration	IR (per 1,000)	Model 1	Model 2	Model 3	Model 4	Model 5
− 18.5	147,834	568	1598694.81	0.35529	1.207 (1.107, 1.316)	1.541 (1.413, 1.681)	1.5 (1.375, 1.636)	1.459 (1.333, 1.597)	1.436 (1.311, 1.572)
18.5–23	1,573,660	5124	17400391.93	0.29448	1 (ref.)	1 (ref.)	1 (ref.)	1 (ref.)	1 (ref.)
23–25	998,895	3014	11080807.63	0.272	0.924 (0.883, 0.966)	0.764 (0.73, 0.799)	0.772 (0.738, 0.808)	0.788 (0.75, 0.828)	0.794 (0.756, 0.834)
25–30	1,180,895	3462	13107037.03	0.26413	0.897 (0.859, 0.936)	0.723 (0.693, 0.755)	0.716 (0.685, 0.748)	0.745 (0.703, 0.79)	0.755 (0.712, 0.8)
30 -	143,797	341	1595614.5	0.21371	0.726 (0.651, 0.81)	0.708 (0.635, 0.79)	0.653 (0.585, 0.73)	0.706 (0.619, 0.805)	0.713 (0.625, 0.813)

Hazard ratios of suicidal death with respect to BMI intervals were examined after adjusting for variables using Multivariable Cox proportional hazard regression analysis

Model 1: Unadjusted

Model 2: Age and Sex

Model 3: Age, Sex, Income, DM, HTN, DYS, Smoke, Drink, Exercise, and Self-abuse

Model 4: Age, Sex, Income, DM, HTN, DYS, Smoke, Drink, Exercise, Self-abuse, and Waist circumference

Model 5: Age, Sex, Income, DM, HTN, DYS, Smoke, Drink, Exercise, Self-abuse, Waist circumference, Schizophrenia, Bipolar disorder, Eating disorder, Cancer, Anxiety, and Substance use disorder

*Abbreviations BMI* Body mass index, *DM* Diabetes mellitus, *HTN* Hypertension, *DYS* Dyslipidemia, *IR* Incidence rate (per 1000)

### Subgroup analysis of suicidal death risk with respect to BMI categories

To further explore how the relationship between suicidal death and BMI varies across different population demographics, we conducted a subgroup analysis (Table 3, S3 Table). The inverse relationship between the risk of suicidal death and BMI was consistent across most population subsets. Notably, individuals with a lower BMI were at an increased risk of suicidal death, irrespective of the presence of MDD (P for interaction 0.58) or their living arrangements (whether in single or multiple-occupancy households, P for interaction 0.21). However, age (*P* = 0.0078) and smoking status (*P* = 0.044) were significant modifiers of the relationship between BMI and the risk of suicidal death. There was a tendency of modest increase in the risk of suicidal death from class I (HR 0.77, 95% CI 0.70–0.86) to class II (HR 0.81, 95% CI 0.66–1.00) obesity. Similarly, in the subgroup of ex-smokers, the risk of suicidal death modestly increased from class I (HR 0.68, 95% CI 0.61–0.76) to class II (HR 0.75, 95% CI 0.57–0.99) obesity. We also estimated the risk of suicidal death associated with each variable. Schizophrenia (HR 5.879, 95% CI 4.88–7.08), bipolar disorder (HR 4.25, 95% CI 3.36–5.36), and substance use disorder (HR 3.65, 95% CI 3.17–4.20) were strongly associated with an increased risk of suicidal death (S4 Table). However, the inverse correlation between BMI and suicidal death remained consistent regardless of the presence of schizophrenia, bipolar disorder, or substance use disorder (S3 Table). Overall, our subgroup analysis corroborates the inverse relationship between BMI and suicidal death across various baseline characteristics.

**Table 3:** Subgroup analysis of suicidal death risk with respect to BMI categories

	BMI	*N*	Suicide	Duration	IR (per 1,000)	Adjusted HR	*P* for interaction
Male	− 18.5	49,168	385	505906.23	0.76101	1.442 (1.294, 1.608)	0.4195
	18.5–23	748,691	3753	8181012.82	0.45875	1 (ref.)	
	23–25	600,857	2403	6638756.22	0.36197	0.809 (0.766, 0.855)	
	25–30	748,329	2739	8292529.17	0.3303	0.76 (0.713, 0.809)	
	30 -	81,033	256	899559.33	0.28458	0.749 (0.647, 0.867)	
Female	− 18.5	98,666	183	1092788.58	0.16746	1.413 (1.209, 1.652)	
	18.5–23	824,969	1371	9219379.12	0.14871	1 (ref.)	
	23–25	398,038	611	4442051.41	0.13755	0.744 (0.675, 0.82)	
	25–30	432,566	723	4814507.86	0.15017	0.745 (0.674, 0.822)	
	30 -	62,764	85	696055.16	0.12212	0.623 (0.495, 0.785)	

Hazard ratios of completed suicide with respect to BMI intervals were examined after adjusting for variables using Multivariable Cox proportional hazard regression analysis. (Age, Sex, Income, DM, HTN, DYS, Smoke, Drink, Exercise, Self-abuse, Waist circumference, Schizophrenia, Bipolar disorder, Eating disorder, Cancer, Anxiety, and Substance use disorder)

*Abbreviations BMI* Body mass index, *IR* Incidence rate (per 1000), *HR* Hazard Ratio

## Discussion

In this study, we provide evidence of an inverse correlation between BMI and the risk of suicidal death. Being underweight was associated with a 1.44-fold increase in the suicidal death risk. Conversely, being class I obesity was associated with a 0.76-fold suicidal death risk, and being class II obesity was associated with a 0.71-fold risk, compared with the suicidal death risk in the normal-weight population. This trend persisted across various population subgroups and was independent of the presence of MDD and the type of living arrangements.

Obesity is considered a risk factor for various adverse health outcomes, including cardiovascular diseases, cancer, and mental disorders [27–30]. Especially, obesity has been linked to a 1.55-fold increase in the risk of major depressive disorder [13].Despite the high prevalence of MDD in obese population, our results suggest that obese individuals have a lower suicidal death risk than those with normal weight. Our findings can be explained by several hypotheses based on previous literature. First, a reduced serotonin level in the central nervous system of individuals with obesity could have contributed to the decreased risk of suicidal death [31]. Insulin resistance, a common feature of obesity, could increase the levels of serum-free fatty acids, competing with tryptophan for binding to serum albumin. As a result, free tryptophan levels can be elevated. Since free tryptophan is essential for serotonin synthesis, insulin resistance might lead to increased serotonin levels in the central nervous system [32, 33]. Low serotonin level in the central nervous system was suggested to associate with increased impulsive behavior, providing possible explanation for lower risk of suicidal death in obese individuals. Second, obesity-induced leptin resistance may have contributed to decreased impulsivity. Leptin, a hormone produced by fat tissue, signals the brain to regulate hunger and energy balance. Obesity can lead to leptin resistance, where the brain becomes less responsive to leptin, contributing to overeating and weight gain. Interestingly, this leptin resistance might also affect brain functions related to impulsivity, with some research suggesting it could paradoxically decrease impulsivity in certain contexts by altering reward processing pathways in the brain [31, 34, 35]. Third, obesity may limit the methods by which individuals choose to end their lives. People from different BMI are tend to choose different suicidal methods. For example, obese individuals being more likely choose intoxication but less likely to opt for hanging, drowning, or jumping, compared with those with normal weight [36]. For obese individuals may have higher physical tolerability to toxic medications, this could have contributed to the lower suicidal death risk in obese individuals. The prevalence of obesity has been steadily increasing since the early 2000s. As a result, our findings differ from recent epidemiological trends regarding obesity and suicide mortality rates. We believe that BMI is one of several factors contributing to suicidal death risk. Future research is needed to better understand the relationship between the rising rates of obesity and suicide mortality.

Our study also highlights the elevated risk of suicide death among underweight individuals, which aligns with the epidemiological characteristics of South Korea, a country with one of the highest suicide rates among Organization for Economic Co-operation and Development nations and a high prevalence of underweight individuals (2.64-fold higher prevalence compared with the U.S.) [37, 38]. We speculate that this subpopulation is the group of interest that deserve attention for health-administrative intervention. Psychological factors such as body image, victimization and bullying, eating disorders, and sleep problems could have contributed to the relationship between suicidal risk and low body weight. Furthermore, neurobiological factors could have contributed to the increased suicidal risk in underweight population although further research should be explored in the future [39]. Decreased muscle mass has been linked with the risk of suicide [40, 41]. Investigating whether systematic interventions such as increasing muscle mass in the underweight population can reduce the suicide risk would be an intriguing area for future research.

Although several previous large-scale cohort studies have explored the relationship between BMI and the risk of suicidal death, our study had several strengths that address the limitations of these studies. McCarthy et al. retrospectively evaluated the Veterans Health Administration National Patient Care Database, which enrolled 4,005,640 individuals and 10,169 suicidal cases [22]. However, the enrolled participants were mainly veterans who were subjected to unusual types of stress and were predominantly male (female, 5.30%). Therefore, the characteristics of the study population limit the generalizability of the results. Bjerkeset et al. analyzed a prospective cohort of 74,332 Norwegian individuals enrolled from 1984 to 86 and followed up until 2002 (HUNT study) [42]. This study demonstrated a 0.82-fold decrease in the risk of suicidal death with an one standard deviation increase in BMI. However, this study was performed about 20 years ago and were based on Western population. Furthermore, small number of suicidal death event (183 cases of suicides including only 1 suicide in underweight group) was included in this study. Elovainio et al. suggested a 2.48-fold increase in the risk of suicidal death among the obese population compared to those of normal weight (Whitehall study) [18]. This research was based on 18,784 men from London aged 40 to 69, registered between 1967 and 70, and followed up for 38 years. Jee et al. suggested an inverse correlation between obesity and suicidal death in a Korean Cancer Prevention Study cohort that included 472 suicide cases [43]. Our study analyzed 4,234,415 individuals, with a median follow-up period of 11.31 years (interquartile range: 11.11–11.57), during which 12,509 suicidal death events were recorded. Our participants were enrolled from the Korean NHID, which is based on the NHIS—a single public health insurance system that requires registration from all Koreans. Given the comprehensive coverage of the NHID and the substantial number of participants and primary events in our study, we believe our data accurately reflects the general Korean population. Taking advantage of this large and representative cohort, we strongly suggest an inverse correlation between BMI and suicidal death events in the Asian population.

Our data clearly demonstrate that the inverse correlation between BMI and suicidal death is consistent regardless of the presence of MDD or the type of living arrangements. These results challenge conventional stereotype that the relationship between BMI and suicidal risk could be influenced by non-biological confounding factors [20, 44, 45]. Our data emphasize the importance of considering physiological mechanisms associated with BMI in mental health vulnerabilities and suicide risk. Further research revealing these biological pathways to establish preventive strategies and interventions aimed at mitigating suicide risk would be an interesting subject to explore. Additionally, our data suggests that special attention for suicidal prevention strategies should be given to the non-MDD or multi-household underweight population, as they are at a high risk of suicidal deaths.

Nonetheless, this study had some unaddressed limitations. First, our study could not establish a causality between BMI and suicidal deaths. Based on a large volume of consistent findings from previous studies, obesity may be directly or indirectly associate with an increase in mental illnesses, but it does not directly influence the suicidal risk. However, our data indicate that patients with a low BMI are especially at a high risk of suicidal deaths, providing strong evidence supporting the need to invest limited administrative resources. Second, we did not account for pharmacological influences such as medications affecting both body weight and suicide risk. The use of antidepressants (e.g., amitriptyline bupropion) [46], anti-psychotics (e.g., olanzapine clozapine) [47], anti-obesity drugs (e.g., Phentermine, Phentermine-topiramate), which have the potential to affect both body weight and risk of suicide, may have influenced the results of our study. However, the substantial sample size and number of suicidal events suggest that this limitation is unlikely to compromise the validity of our conclusions. Third, while we made our best efforts to adjust for potential confounding variables such as exercise, income, and self-harm, we acknowledge that some confounding factors may still remain. Additionally, cultural and lifestyle differences between groups could have influenced the results. Fourth, we did not measure changes in body weight during the follow-up period, nor did we assess the participants’ body composition. The association of body weight changes or body composition components with the suicidal death risk should be explored in future studies.

## Conclusion

In summary, our study provides robust evidence for an inverse correlation between obesity and suicidal death. Individuals with low BMI are at a high risk of committing suicide. Future studies evaluating whether targeted interventions in these subpopulations can reduce the risk of suicidal death would enhance the value of our study.

## Electronic supplementary material

Below is the link to the electronic supplementary material.

Supplementary Material 1: Figure S1. Enrollment scheme of study participants.

Supplementary Material 2: Table S1. Correlation Analysis of Individual Variables. Table S2. Suicide risk based on obesity presence as defined by waist circumference or BMI. Table S3. Subgroup analysis of suicidal death risk with respect to BMI categories. Table S4. The risk of suicidal death with respect to each variable.

## Data Availability

The National Health Insurance Data Integration Service website of the “Health Insurance Corporation” provided the data used in this study (http://nhiss.nhis.or.kr/bd/ab/bdaba021eng.do). Petitioners must submit completed application forms, study plans, and IRB-approved paperwork to the NHIS study Grant consideration Board for consideration in order to be granted access to NHIS data.

## Abbreviations

BMI: Body Mass Index
CI: Confidence Interval
CKD: Chronic Kidney Disease
DBP: Diastolic Blood Pressure
DM: Diabetes Mellitus
DYS: Dyslipidemia
eGFR: Estimated Glomerular Filtration Rate
HDL-C: High-Density Lipoprotein Cholesterol
HR: Hazard Ratio
HTN: Hypertension
ICD: International Classification of Disorders
IR: Incidence Rate
LDL-C: Low-Density Lipoprotein Cholesterol
MDD: Major Depressive Disorder
NHID: National Health Information Database
NHIS: National Health Insurance Service
SBP: Systolic Blood Pressure
