# Supplementary figures and images for: Inverse association between obesity and suicidal death risk
Source: BMC Psychiatry. 2025 Jan 8;25:27. doi: 10.1186/s12888-024-06381-z (PMC11714859; doi:10.1186/s12888-024-06381-z)

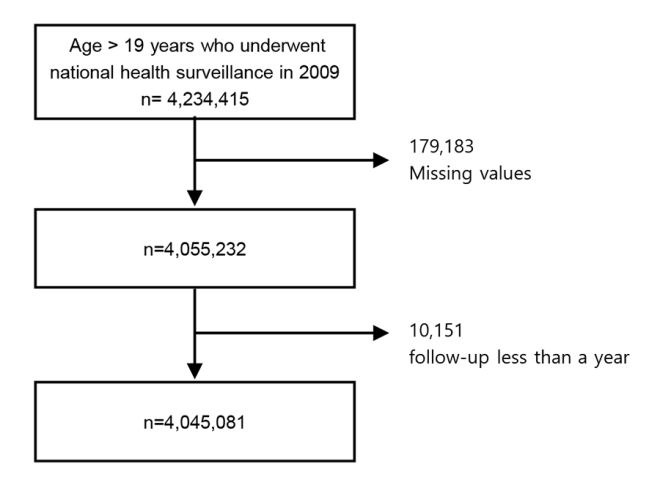


**Figure S1.** Enrollment scheme of study participants

Supplement: Supplementary file 1 — Supplementary Material 1: Figure S1. Enrollment scheme of study participants. [file 12888_2024_6381_MOESM1_ESM.docx]
