# Supplementary material for: Inverse association between obesity and suicidal death risk
Source: BMC Psychiatry. 2025 Jan 8;25:27. doi: 10.1186/s12888-024-06381-z (PMC11714859; doi:10.1186/s12888-024-06381-z)
Supplement: Supplementary file 2 — Supplementary Material 2: Table S1. Correlation Analysis of Individual Variables. Table S2. Suicide risk based on obesity presence as defined by waist circumference or BMI. Table S3. Subgroup analysis of suicidal death risk with respect to BMI categories. Table S4. The risk of suicidal death with respect to each variable. [file 12888_2024_6381_MOESM2_ESM.docx]

**Supplementary Table**

**Table S1.** Correlation Analysis of Individual Variables

**Table S2.** Suicide risk based on obesity presence as defined by waist circumference or BMI.

**Table S3.** Subgroup analysis of suicidal death risk with respect to BMI categories

**Table S4.** The risk of suicidal death with respect to each variable.

**Table S1.** Correlation Analysis of Individual Variables

|  | Variance Inflation Factor |
| --- | --- |
| BMI | 2.37202 |
| Age | 1.43549 |
| Sex | 2.015 |
| Income | 1.03605 |
| DM | 1.09979 |
| HP | 1.27921 |
| DYS | 1.10577 |
| Smoke | 1.70077 |
| Drink | 1.34999 |
| Exercise | 1.01373 |
| Self-abuse | 1.00007 |
| Waist circum. | 2.99983 |
| Schizo. | 1.00315 |
| Bipolar | 1.00326 |
| Eating disorder | 1.00119 |
| Cancer | 1.00979 |
| Anxiety | 1.00731 |
| Substance use disorder | 1.00413 |

**Table S2. Suicide risk based on obesity presence as defined by waist circumference or BMI.**

|  | N | Suicide | Duration | IR (per 1,000) | Model 1 | Model 2 | Model 3 | P for interaction |
| --- | --- | --- | --- | --- | --- | --- | --- | --- |
| Abdominal obes. (-) & BMI < 25 | 2593429 | 8151 | 28728019.14 | 0.28373 | 1 (ref.) | 1 (ref.) | 1 (ref.) | 0.6965 |
| Abdominal obes. (-) & BMI ≥ 25 | 656779 | 1776 | 7339641.88 | 0.24197 | 0.853 (0.81, 0.898) | 0.781 (0.742, 0.822) | 0.788 (0.748, 0.829) |  |
| Abdominal obes. (+) & BMI < 25 | 126960 | 555 | 1351875.23 | 0.41054 | 1.445 (1.326, 1.575) | 1.02 (0.936, 1.113) | 0.986 (0.904, 1.076) |  |
| Abdominal obes. (+) & BMI ≥ 25 | 667913 | 2027 | 7363009.65 | 0.2753 | 0.97 (0.924, 1.018) | 0.788 (0.75, 0.827) | 0.76 (0.723, 0.799) |  |

Hazard ratios of completed suicide with respect to obesity (BMI, abdominal) were examined after adjusting for variables using Multivariable Cox proportional hazard regression analysis. Model 1: Unadjusted, Model 2: Age, Sex, Model 3: Age, Sex, Income, DM, HP, DYS, Smoke, Drink, Exercise, Self-abuse. BMI: Body mass index, IR: Incidence rate (per 1000).

**Table S3.** Subgroup analysis of suicidal death risk with respect to BMI categories

|  | **BMI** | **N** | **Suicide** | **Duration** | **IR (per 1,000)** | **Adjusted HR** | ***P* for interaction** |
| --- | --- | --- | --- | --- | --- | --- | --- |
| **< 40** | - 18.5 | 85649 | 167 | 967629.41 | 0.17259 | 1.313 (1.115, 1.547) | 0.0109 |
|  | 18.5 - 23 | 574040 | 1199 | 6487852.22 | 0.18481 | 1 (ref.) |  |
|  | 23 - 25 | 258458 | 562 | 2920444.84 | 0.19244 | 0.815 (0.735, 0.903) |  |
|  | 25 - 30 | 302065 | 673 | 3407929.54 | 0.19748 | 0.779 (0.703, 0.864) |  |
|  | 30 - | 51786 | 119 | 582580.32 | 0.20426 | 0.826 (0.674, 1.012) |  |
| **40 - 64** | - 18.5 | 43032 | 239 | 471956.04 | 0.5064 | 1.623 (1.418, 1.858) |  |
|  | 18.5 - 23 | 816739 | 2596 | 9136093.55 | 0.28415 | 1 (ref.) |  |
|  | 23 - 25 | 603661 | 1633 | 6764639.65 | 0.2414 | 0.778 (0.729, 0.83) |  |
|  | 25 - 30 | 709990 | 1907 | 7949478.89 | 0.23989 | 0.744 (0.693, 0.798) |  |
|  | 30 - | 74523 | 151 | 832208.46 | 0.18144 | 0.625 (0.523, 0.748) |  |
| **≥ 65** | - 18.5 | 19153 | 162 | 159109.35 | 1.01817 | 1.226 (1.039, 1.447) |  |
|  | 18.5 - 23 | 182881 | 1329 | 1776446.17 | 0.74812 | 1 (ref.) |  |
|  | 23 - 25 | 136776 | 819 | 1395723.13 | 0.58679 | 0.851 (0.778, 0.931) |  |
|  | 25 - 30 | 168840 | 882 | 1749628.6 | 0.50411 | 0.809 (0.736, 0.889) |  |
|  | 30 - | 17488 | 71 | 180825.72 | 0.39264 | 0.783 (0.61, 1.005) |  |
| **MDD (-)** | - 18.5 | 143353 | 508 | 154770.45 | 3.28228 | 1.443 (1.313, 1.586) | 0.7007 |
|  | 18.5 - 23 | 1524818 | 4616 | 16884442.29 | 0.27339 | 1 (ref.) |  |
|  | 23 - 25 | 965247 | 2711 | 10718892.21 | 0.25292 | 0.797 (0.757, 0.839) |  |
|  | 25 - 30 | 1140319 | 3118 | 12669222.36 | 0.24611 | 0.76 (0.716, 0.806) |  |
|  | 30 - | 138849 | 299 | 1542302.67 | 0.19387 | 0.708 (0.617, 0.812) |  |
| **MDD (+)** | - 18.5 | 4481 | 60 | 43924.36 | 1.36598 | 1.278 (0.956, 1.709) |  |
|  | 18.5 - 23 | 48842 | 508 | 515949.64 | 0.98459 | 1 (ref.) |  |
|  | 23 - 25 | 33648 | 303 | 361915.42 | 0.83721 | 0.796 (0.684, 0.926) |  |
|  | 25 - 30 | 40576 | 344 | 437814.67 | 0.78572 | 0.772 (0.665, 0.895) |  |
|  | 30 - | 4948 | 42 | 53311.82 | 0.78782 | 0.875 (0.621, 1.232) |  |
| **Multi-person household** | - 18.5 | 90961 | 424 | 963737.05 | 0.43995 | 1.499 (1.349, 1.666) | 0.1913 |
|  | 18.5 - 23 | 1128071 | 3860 | 12413711.35 | 0.31095 | 1 (ref.) |  |
|  | 23 - 25 | 783968 | 2370 | 8681459.75 | 0.273 | 0.792 (0.749, 0.837) |  |
|  | 25 - 30 | 945994 | 2743 | 10487284.73 | 0.26155 | 0.755 (0.709, 0.804) |  |
|  | 30 - | 110129 | 249 | 1220526.9 | 0.20401 | 0.686 (0.591, 0.796) |  |
| **Single-person household** | - 18.5 | 56873 | 144 | 634957.76 | 0.22679 | 1.244 (1.045, 1.48) |  |
|  | 18.5 - 23 | 445589 | 1264 | 4986680.58 | 0.25348 | 1 (ref.) |  |
|  | 23 - 25 | 214927 | 644 | 2399347.88 | 0.26841 | 0.807 (0.733, 0.89) |  |
|  | 25 - 30 | 234901 | 719 | 2619752.29 | 0.27445 | 0.768 (0.695, 0.849) |  |
|  | 30 - | 33668 | 92 | 375087.6 | 0.24528 | 0.798 (0.638, 0.999) |  |
| **Smoke, non** | - 18.5 | 104041 | 237 | 1140852.31 | 0.20774 | 1.406 (1.227, 1.612) | 0.0631 |
|  | 18.5 - 23 | 1005366 | 2163 | 11178879.84 | 0.19349 | 1 (ref.) |  |
|  | 23 - 25 | 568864 | 1224 | 6324400.78 | 0.19354 | 0.789 (0.733, 0.849) |  |
|  | 25 - 30 | 641015 | 1470 | 7116647.82 | 0.20656 | 0.79 (0.732, 0.854) |  |
|  | 30 - | 81875 | 143 | 907012.76 | 0.15766 | 0.694 (0.578, 0.835) |  |
| **Smoke, ex** | - 18.5 | 10162 | 71 | 103489.05 | 0.68606 | 1.79 (1.401, 2.288) |  |
|  | 18.5 - 23 | 175826 | 754 | 1919106.89 | 0.39289 | 1 (ref.) |  |
|  | 23 - 25 | 165237 | 536 | 1825727.28 | 0.29358 | 0.726 (0.649, 0.812) |  |
|  | 25 - 30 | 210904 | 650 | 2335463.23 | 0.27832 | 0.693 (0.62, 0.774) |  |
|  | 30 - | 19394 | 58 | 214824.21 | 0.26999 | 0.77 (0.584, 1.015) |  |
| **Smoking, current** | - 18.5 | 33631 | 260 | 354353.45 | 0.73373 | 1.394 (1.222, 1.589) |  |
|  | 18.5 - 23 | 392468 | 2207 | 4302405.2 | 0.51297 | 1 (ref.) |  |
|  | 23 - 25 | 264794 | 1254 | 2930679.57 | 0.42789 | 0.828 (0.77, 0.89) |  |
|  | 25 - 30 | 328976 | 1342 | 3654925.98 | 0.36718 | 0.746 (0.69, 0.807) |  |
|  | 30 - | 42528 | 140 | 473777.53 | 0.2955 | 0.71 (0.589, 0.856) |  |
| **Drink, non** | - 18.5 | 84083 | 279 | 901470.48 | 0.30949 | 1.398 (1.231, 1.587) | 0.0378 |
|  | 18.5 - 23 | 840765 | 2325 | 9259938.13 | 0.25108 | 1 (ref.) |  |
|  | 23 - 25 | 502183 | 1282 | 5543877.91 | 0.23125 | 0.784 (0.73, 0.842) |  |
|  | 25 - 30 | 579253 | 1548 | 6394498.45 | 0.24208 | 0.795 (0.737, 0.857) |  |
|  | 30 - | 74552 | 143 | 822839.43 | 0.17379 | 0.664 (0.553, 0.799) |  |
| **Drink, mild** | - 18.5 | 57095 | 229 | 628767.96 | 0.3642 | 1.531 (1.333, 1.759) |  |
|  | 18.5 - 23 | 633569 | 2128 | 7058971.27 | 0.30146 | 1 (ref.) |  |
|  | 23 - 25 | 413780 | 1314 | 4620398.62 | 0.28439 | 0.811 (0.755, 0.871) |  |
|  | 25 - 30 | 483531 | 1440 | 5399919.65 | 0.26667 | 0.751 (0.696, 0.812) |  |
|  | 30 - | 53973 | 150 | 602836.38 | 0.24882 | 0.824 (0.688, 0.988) |  |
| **Drink, heavy** | - 18.5 | 6656 | 60 | 68456.37 | 0.87647 | 1.301 (0.998, 1.696) |  |
|  | 18.5 - 23 | 99326 | 671 | 1081482.53 | 0.62044 | 1 (ref.) |  |
|  | 23 - 25 | 82932 | 418 | 916531.1 | 0.45607 | 0.768 (0.678, 0.869) |  |
|  | 25 - 30 | 118111 | 474 | 1312618.92 | 0.36111 | 0.65 (0.574, 0.736) |  |
|  | 30 - | 15272 | 48 | 169938.68 | 0.28245 | 0.585 (0.432, 0.791) |  |
| **Regular exercise (-)** | - 18.5 | 133491 | 483 | 1445056.02 | 0.33424 | 1.389 (1.26, 1.532) | 0.2022 |
|  | 18.5 - 23 | 1314128 | 4205 | 14524668.87 | 0.28951 | 1 (ref.) |  |
|  | 23 - 25 | 797871 | 2333 | 8845975.88 | 0.26374 | 0.778 (0.737, 0.821) |  |
|  | 25 - 30 | 945076 | 2691 | 10483690.82 | 0.25668 | 0.741 (0.697, 0.789) |  |
|  | 30 - | 118878 | 278 | 1318060.36 | 0.21092 | 0.707 (0.613, 0.815) |  |
| **Regular exercise (+)** | - 18.5 | 14343 | 85 | 153638.79 | 0.55325 | 1.731 (1.384, 2.165) |  |
|  | 18.5 - 23 | 259532 | 919 | 2875723.06 | 0.31957 | 1 (ref.) |  |
|  | 23 - 25 | 201024 | 681 | 2234831.75 | 0.30472 | 0.86 (0.778, 0.952) |  |
|  | 25 - 30 | 235819 | 771 | 2623346.21 | 0.2939 | 0.811 (0.731, 0.898) |  |
|  | 30 - | 24919 | 63 | 277554.14 | 0.22698 | 0.735 (0.564, 0.958) |  |
| **Cancer (-)** | - 18.5 | 145853 | 553 | 1580488.08 | 0.34989 | 1.443 (1.317, 1.581) | 0.2491 |
|  | 18.5 - 23 | 1552730 | 4994 | 17187974.66 | 0.29055 | 1 (ref.) |  |
|  | 23 - 25 | 985909 | 2963 | 10946924.23 | 0.27067 | 0.799 (0.76, 0.839) |  |
|  | 25 - 30 | 1166724 | 3410 | 12960774.78 | 0.2631 | 0.759 (0.716, 0.805) |  |
|  | 30 - | 142330 | 335 | 1580373.35 | 0.21198 | 0.712 (0.624, 0.814) |  |
| **Cancer (+)** | - 18.5 | 1981 | 15 | 18206.73 | 0.82387 | 1.188 (0.696, 2.028) |  |
|  | 18.5 - 23 | 20930 | 130 | 212417.27 | 0.612 | 1 (ref.) |  |
|  | 23 - 25 | 12986 | 51 | 133883.4 | 0.38093 | 0.594 (0.429, 0.821) |  |
|  | 25 - 30 | 14171 | 52 | 146262.25 | 0.35553 | 0.559 (0.404, 0.773) |  |
|  | 30 - | 1467 | 6 | 15241.15 | 0.39367 | 0.759 (0.333, 1.725) |  |
| **Schizophrenia (-)** | - 18.5 | 147657 | 560 | 1596999.02 | 0.35066 | 1.429 (1.305, 1.565) | 0.2903 |
|  | 18.5 - 23 | 1572086 | 5095 | 17382956.01 | 0.2931 | 1 (ref.) |  |
|  | 23 - 25 | 997596 | 2989 | 11066454.5 | 0.2701 | 0.793 (0.755, 0.833) |  |
|  | 25 - 30 | 1178769 | 3415 | 13083680.36 | 0.26101 | 0.752 (0.709, 0.797) |  |
|  | 30 - | 143322 | 334 | 1590447.3 | 0.21 | 0.712 (0.624, 0.813) |  |
| **Schizophrenia (+)** | - 18.5 | 177 | 8 | 1695.79 | 4.71757 | 2.314 (1.057, 5.065) |  |
|  | 18.5 - 23 | 1574 | 29 | 17435.92 | 1.66323 | 1 (ref.) |  |
|  | 23 - 25 | 1299 | 25 | 14353.13 | 1.74178 | 0.975 (0.571, 1.665) |  |
|  | 25 - 30 | 2126 | 47 | 23356.67 | 2.01227 | 1.242 (0.781, 1.977) |  |
|  | 30 - | 475 | 7 | 5167.2 | 1.3547 | 0.948 (0.414, 2.17) |  |
| **Bipolar disorder (-)** | - 18.5 | 147745 | 568 | 1597777.05 | 0.35549 | 1.444 (1.319, 1.581) | 0.2498 |
|  | 18.5 - 23 | 1572558 | 5101 | 17388758.88 | 0.29335 | 1 (ref.) |  |
|  | 23 - 25 | 997996 | 3001 | 11071328.81 | 0.27106 | 0.795 (0.756, 0.835) |  |
|  | 25 - 30 | 1179478 | 3428 | 13092015.07 | 0.26184 | 0.753 (0.71, 0.798) |  |
|  | 30 - | 143495 | 337 | 1592365.54 | 0.21163 | 0.713 (0.625, 0.814) |  |
| **Bipolar disorder (+)** | - 18.5 | 89 | 0 | 917.76 | 0 | - |  |
|  | 18.5 - 23 | 1102 | 23 | 11633.05 | 1.97713 | 1 (ref.) |  |
|  | 23 - 25 | 899 | 13 | 9478.82 | 1.37148 | 0.678 (0.343, 1.339) |  |
|  | 25 - 30 | 1417 | 34 | 15021.96 | 2.26335 | 1.218 (0.716, 2.073) |  |
|  | 30 - | 302 | 4 | 3248.96 | 1.23116 | 0.762 (0.262, 2.21) |  |
| **Eating disorder (-)** | - 18.5 | 147748 | 567 | 1597807.93 | 0.35486 | 1.434 (1.31, 1.57) | 0.3659 |
|  | 18.5 - 23 | 1573031 | 5123 | 17394374.28 | 0.29452 | 1 (ref.) |  |
|  | 23 - 25 | 998595 | 3011 | 11077163.16 | 0.27182 | 0.793 (0.755, 0.833) |  |
|  | 25 - 30 | 1180541 | 3459 | 13102930.55 | 0.26399 | 0.754 (0.712, 0.8) |  |
|  | 30 - | 143739 | 340 | 1594965.06 | 0.21317 | 0.711 (0.623, 0.811) |  |
| **Eating disorder (+)** | - 18.5 | 86 | 1 | 886.88 | 1.12755 | 8.112 (0.513, 128.412) |  |
|  | 18.5 - 23 | 629 | 1 | 6017.65 | 0.16618 | 1 (ref.) |  |
|  | 23 - 25 | 300 | 3 | 3644.47 | 0.82316 | 5.038 (0.53, 47.891) |  |
|  | 25 - 30 | 354 | 3 | 4106.48 | 0.73055 | 4.661 (0.49, 44.32) |  |
|  | 30 - | 58 | 1 | 649.44 | 1.53979 | 11.985 (0.762, 188.552) |  |
| **Anxiety disorder (-)** | - 18.5 | 146282 | 545 | 1583049.17 | 0.34427 | 1.429 (1.303, 1.567) | 0.711 |
|  | 18.5 - 23 | 1556035 | 4953 | 17209619.74 | 0.2878 | 1 (ref.) |  |
|  | 23 - 25 | 987108 | 2920 | 10952046.88 | 0.26662 | 0.796 (0.757, 0.836) |  |
|  | 25 - 30 | 1167551 | 3366 | 12960896.08 | 0.2597 | 0.758 (0.715, 0.804) |  |
|  | 30 - | 142316 | 333 | 1579500.95 | 0.21083 | 0.717 (0.628, 0.819) |  |
| **Anxiety disorder (+)** | - 18.5 | 1552 | 23 | 15645.64 | 1.47006 | 1.618 (1.047, 2.503) |  |
|  | 18.5 - 23 | 17625 | 171 | 190772.19 | 0.89636 | 1 (ref.) |  |
|  | 23 - 25 | 11787 | 94 | 128760.75 | 0.73004 | 0.741 (0.576, 0.954) |  |
|  | 25 - 30 | 13344 | 96 | 146140.95 | 0.6569 | 0.658 (0.511, 0.847) |  |
|  | 30 - | 1481 | 8 | 16113.55 | 0.49648 | 0.577 (0.283, 1.177) |  |
| **Substance use disorder (-)** | - 18.5 | 147331 | 558 | 1594416.35 | 0.34997 | 1.452 (1.325, 1.591) | 0.1729 |
|  | 18.5 - 23 | 1569568 | 5020 | 17360955.63 | 0.28915 | 1 (ref.) |  |
|  | 23 - 25 | 996697 | 2965 | 11057901.44 | 0.26813 | 0.793 (0.755, 0.834) |  |
|  | 25 - 30 | 1178297 | 3424 | 13080011.15 | 0.26177 | 0.758 (0.715, 0.803) |  |
|  | 30 - | 143524 | 334 | 1592813.55 | 0.20969 | 0.708 (0.62, 0.808) |  |
| **Substance use disorder (+)** | - 18.5 | 503 | 10 | 4278.46 | 2.33729 | 0.874 (0.456, 1.672) |  |
|  | 18.5 - 23 | 4092 | 104 | 39436.3 | 2.63716 | 1 (ref.) |  |
|  | 23 - 25 | 2198 | 49 | 22906.19 | 2.13916 | 0.854 (0.608, 1.2) |  |
|  | 25 - 30 | 2598 | 38 | 27025.88 | 1.40606 | 0.588 (0.405, 0.855) |  |
|  | 30 - | 273 | 7 | 2800.95 | 2.49915 | 1.125 (0.521, 2.429) |  |

Hazard ratios of completed suicide with respect to BMI intervals were examined after adjusting for variables using Multivariable Cox proportional hazard regression analysis. Adjusted Hazard ratio was estimated after adjusting for Age, Sex, Income, DM, HTN, DYS, Smoke, Drink, Exercise, Self-abuse, Waist circumference, Schizophrenia, Bipolar disorder, Eating disorder, Cancer, Anxiety, and Substance use disorder.

*Abbreviations:* *BMI* Body mass index, *MDD* Major depressive disorder, *IR* Incidence rate (per 1000), *HR* Hazard Ratio.

**Table S4.** The risk of suicidal death with respect to each variable.

|  | Adjusted HR |
| --- | --- |
| **Age, per 1** | 1.036 (1.035, 1.038) |
| **Sex, female** | 0.39 (0.369, 0.412) |
| **Income** |  |
| Q1 | 1 (ref.) |
| Q2 | 0.944 (0.897, 0.995) |
| Q3 | 0.833 (0.792, 0.875) |
| Q4 | 0.727 (0.692, 0.764) |
| **DM** | 1.217 (1.154, 1.284) |
| **Hypertension** | 1.186 (1.138, 1.236) |
| **Dyslipidemia** | 0.991 (0.946, 1.038) |
| **Smoke** |  |
| Non | 1 (ref.) |
| Ex | 0.981 (0.926, 1.039) |
| Current | 1.616 (1.54, 1.696) |
| **Drink** |  |
| Non | 1 (ref.) |
| Mild | 1.012 (0.97, 1.055) |
| Heavy | 1.284 (1.21, 1.363) |
| **Regular exercise** | 1.013 (0.969, 1.058) |
| **Waist circum., per 1** | 0.996 (0.992, 0.999) |
| **Schizophrenia** | 5.879 (4.881, 7.082) |
| **Bipolar** | 4.246 (3.363, 5.36) |
| **Eating disorder** | 1.475 (0.766, 2.837) |
| **Cancer** | 1.459 (1.287, 1.654) |
| **Anxiety** | 2.553 (2.306, 2.827) |
| **Substance use disorder** | 3.648 (3.172, 4.195) |

Hazard ratios of completed suicide with respect to each variable were examined by using Multivariable Cox proportional hazard regression analysis. Age, Sex, Income, DM, HTN, DYS, Smoke, Drink, Exercise, Self-abuse, Waist circumference, Schizophrenia, Bipolar disorder, Eating disorder, Cancer, Anxiety, and Substance use disorder were adjusted for estimating hazard ratios.

*Abbreviations:* *DM* Diabetes mellitus, *IR* Incidence rate (per 1000), *HR* Hazard Ratio.
